# Elevated Troponin T (TnT) in Non-acute Coronary Syndrome (ACS) Due to Dermatomyositis

**DOI:** 10.7759/cureus.75692

**Published:** 2024-12-14

**Authors:** Abdulaziz S Alnakhli, Kawther AlShaikh, Ahad Al Saud, Owais Rahim

**Affiliations:** 1 Emergency Medicine, King Khalid University Hospital, Riyadh, SAU; 2 Cardiology, King Khalid University Hospital, Riyadh, SAU

**Keywords:** acute coronary syndrome, dermatomyositis, high troponin-t, inflammatory myopathies, polymyositis

## Abstract

Troponin is a highly specific biomarker for myocardial injury. It plays a critical role in the diagnosis of acute coronary syndrome (ACS). However, elevated troponin levels are not exclusively due to cardiac ischemia and may be observed in many non-cardiac conditions, including inflammatory myopathies. These scenarios present significant diagnostic challenges, particularly when clinical symptoms and ECG (electrocardiogram) findings do not align with cardiac pathology.

Recognizing alternative etiologies, such as autoimmune diseases, is essential to prevent unnecessary investigations and ensure appropriate management. This report emphasizes the importance of a comprehensive diagnostic approach to identify non-cardiac causes of high troponin levels and to avoid misdiagnosing patients.

## Introduction

According to the European Society of Cardiology and the American College of Cardiology, troponins are the preferred cardiac markers for detecting myocardial injury due to their sensitivity and specificity [1]. For this reason, troponin levels are routinely measured in patients presenting to the emergency department with symptoms such as syncope, dyspnea, chest pain, or suspected myocardial infarction (MI). Although elevated troponin levels indicate myocardial damage, they do not always confirm cardiac ischemia [2,3]. Numerous mechanisms, such as renal failure, sepsis, and myocarditis, can lead to elevated troponin levels. Therefore, relying solely on troponin levels in the presence of a normal ECG (electrocardiogram) can result in unnecessary invasive tests and can be harmful to the patient with incorrect diagnoses. Clinicians must consider the broad range of causes for elevated troponin levels [4]. This report presents a case where a patient exhibited elevated troponin levels without any cardiac involvement, and the diagnosis was ultimately attributed to dermatomyositis a form of inflammatory myopathy. This was considered due to the other clinical findings that match the aforementioned disorder.

## Case presentation

A 57-year-old woman, known to have type 2 diabetes mellitus, hypertension, dyslipidemia, bronchial asthma, and a history of mitral valve repair seven years ago, presented to the emergency department (ED) with complaints of progressive weakness in her upper and lower limbs that started three months ago and have become worse in the past week to the point that it affects her quality of life. It is associated with pain in the posterior aspect of both thighs. She also reported a history of positional dizziness. She denied any chest pain, shortness of breath, palpitations, syncope, dyspnea, fever, abdominal pain, lower limb swelling, wheezing, cough, heartburn, or recent infections.

Her home medications were as follows: aspirin 81 mg, oral, daily; atorvastatin 40 mg, oral at bedtime; bisoprolol, 10 mg, oral, daily; budesonide-formoterol 160 mcg - 4.5 mcg inhaled via aerosol with adapter, 2 puffs, twice a day; Diamicron MR 120 mg, oral, every morning; insulin glargine 15 units, subcutaneous every morning; linagliptine 5 mg, oral, daily; lisinopril 20 mg, oral, three times a day; metformin 500 mg, oral, twice a day; salbutamol 100 mcg puff metered dose inhaler (MDI), 2 puffs every 4 hours as needed.

As the patient has hypertension and a history of mitral valve repair, medications like aspirin are to reduce the risk of cardiovascular disease while bisoprolol is to control the patient's hypertension. Budesonide-formoterol is used to control the patient's asthma.

Admission vitals

Her temperature was 36.5 degrees Celsius, heart rate was 70 beats per minute, blood pressure was 129/95 millimeters of mercury (mmHg), and oxygen saturation was 96% on room air.

Initial investigations

**Figure 1:**
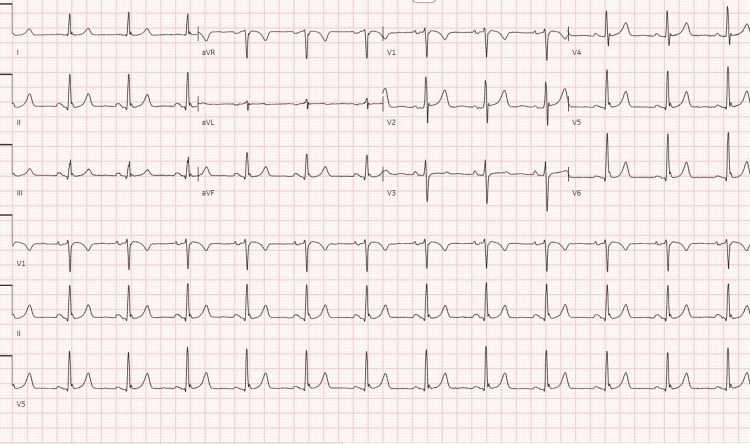
ECG report

The ECG was unremarkable, and she has a normal sinus rhythm with a PR interval of 144 ms (the time between atrial depolarization and ventricular depolarization) and a QRS interval of 90 ms (the time required for a stimulus to spread through the ventricles) with no new nor old ischemic changes (Figure 1).

Table 1 shows the summary of the laboratory results​​.

**Table 1:** Laboratory results

Category	Parameter	Result	Notes
Creatine Kinase (CK)	CK	10,217 U/L	Significantly elevated
Complete Blood Count (CBC)	General	Unremarkable	Normal
	White Blood Cells (WBC)	6.85 x 10⁹/L	Within normal limits
	Hemoglobin (Hgb)	142 g/L	Normal
	Hematocrit (Hct)	45.2%	Normal
	Platelets (Plt)	166 x 10⁹/L	Normal
Renal Function Tests (RFT)	General	Unremarkable	Normal
Electrolytes	Sodium (Na)	138 mmol/L	Normal
	Potassium (K)	3.46 mmol/L	Normal
	Chloride (Cl)	95.8 mmol/L	Normal
Renal Indicators	Creatinine (Cr)	37 μmol/L	Normal
	Blood Urea Nitrogen (BUN)	3.4 mmol/L	Normal
Liver Function Tests	Alanine Aminotransferase (ALT)	443 U/L	Elevated, trending up
	Aspartate Aminotransferase (AST)	316 U/L	Elevated, trending up
Cardiac Markers	Troponin T (TnT)	413 ng/L	Increased from 394 ng/L, possible cardiac injury
	Brain Natriuretic Peptide (BNP)	454 pg/mL	Elevated
Magnesium (Mg)	Magnesium	0.63 mmol/L	Low, requires further evaluation
Thyroid Panel	General	Normal	Normal

The increase in CK levels indicates muscle injury. The trends in alanine aminotransferase (ALT) and aspartate aminotransferase (AST) with liver involvement might suggest myositis though it is not definite. BNP elevation is attributed to the patient's hypertension.

The autoimmune panel came back as follows: antinuclear antibody (ANA) was positive at a titer of 1:160 with a nucleolar pattern suggesting potential autoimmune activity, antineutrophil cytoplasmic antibodies (ANCA) was negative, complement levels - C3 was elevated and C4 was normal, double-stranded DNA (dsDNA) was 9.91, negative; Anti Jo1 was 0.66 negative; and Anti-SSM was 0.96, negative. Interpretation of the positive ANA and the negative DsDNA was suggestive of an autoimmune disease that is less active. Anti Jo1 is a myositis-specific autoantibody commonly found positive in patients with lung involvement and less skin involvement. Anti-SSM is a specific systemic lupus erythematosus (SLE) marker; negative results indicate a lower likelihood of SLE.

Transthoracic Echocardiogram

A two-dimensional transthoracic echocardiogram with imaging modality that describes cardiac motion (M-mode) and Doppler was performed while the patient was in sinus rhythm. Compared to the previous study, no significant changes were noted. Left ventricular size and wall thickness were normal. Systolic function was low-normal, with an estimated left ventricular ejection fraction (LVEF) of 50-55%. Right ventricular size and systolic function were normal. Post bio-prosthetic mitral valve repair, there are moderately elevated transmitral gradients (mean gradient of 9 mmHg) and mild mitral regurgitation. The tricuspid regurgitation signal was insufficient to assess right ventricular systolic pressure (RVSP), but Doppler findings did not indicate pulmonary hypertension. No pericardial effusion or regional wall motion abnormalities were present. The findings of the echocardiogram report did not require additional imaging and contradict the impression of ACS.

*Physical Examination*​​​​​

The patient was sitting comfortably in bed, alert, and oriented to time, place, and person. Chest examination was unremarkable with bilateral air entry with vesicular breath sounds; no added lung sounds were auscultated. Cardiovascular system (CVS), heart sounds, both first and second (S1, S2), were audible with no additional heart sounds or murmurs detected. Abdominal examination revealed that the abdomen was soft and lax with no tenderness or guarding.

Neurological Examination

Table 2 lists the findings of the neurological exam. No gross sensory loss was observed.

**Table 2:** Muscle power assessment

Body Part	Movement	Strength
Shoulders	Bilateral	4/5
Elbows	Flexion (bilateral)	4/5
Elbows	Extension (bilateral)	4/5
Hips	Flexion (bilateral)	3/5
Hips	Extension (bilateral)	3/5
Knees	Flexion (bilateral)	4/5
Knees	Extension (bilateral)	3/5
Feet	Flexion (bilateral)	5/5
Feet	Extension (bilateral)	5/5

Skin Examination

A notable, round, well-defined erythematous patch measuring 1.5 x 2 cm was present on the left side of the chest (approximately in the third intercostal space). It was non-tender with no secondary skin changes and surrounding telangiectasias. Mild skin thickening was noted distal to the proximal interphalangeal (PIP) joints, but no digital ulcers were present on both hands. Two telangiectasias were observed on both sides of the cheeks.

The initial Impression was ACS/non-ST-elevation myocardial infarction (NSTEMI) with an atypical presentation, due to the elevation in troponin levels as well as the history of MV repair and hypertension. Therefore, cardiology was consulted. Due to the lack of any cardiac symptoms and no clinical suspicion of cardiac ischemia while taking the autoimmune panel and the raised creatine kinase (CK) levels under consideration, together with the prominent skin findings that align with Gottron's sign with periungual telangiectasias, further cardiac workup was not warranted. The patient was further evaluated by a rheumatologist. Using Bohan and Peter's criteria, rheumatology reached a diagnosis of dermatomyositis based on the clinical findings and no evidence of cardiac causes. The patient was started on steroids, which significantly improved their symptoms, and then was set on a long-term management plan for dermatomyositis. 

## Discussion

​​​​​This case underlines the importance of considering inflammatory myopathies like dermatomyositis as potential non-coronary causes of troponin elevation. Dermatomyositis is a rare systemic inflammatory disease primarily affecting the skin and muscles, causing progressive muscle weakness, elevated muscle enzymes, e.g. creatine kinase (CK), and sometimes cardiac involvement. High troponin levels in dermatomyositis are not uncommon and can pertain to myocardial inflammation or damage due to the systemic nature of the disease [1].

Troponin T and troponin I exist in both cardiac and skeletal muscles, but they are translated by different genes in each muscle type, making them immunologically distinct from each other. As seen in this case report, patients with inflammatory myopathy present with high troponin T readings, which is initially attributed to an atypical presentation of ACS; however, the current patient had no cardiac symptoms. As we further investigated, looking at the patient's progressive muscle weakness and pain, raised CK levels, skin findings that were synonymous with Gottron's sign, and the autoimmune results, it became evident that the elevated troponin T levels were due to the constant breakdown and destruction of the skeletal muscle. Similar studies suggest that in such scenarios assays for measuring troponin I levels should be introduced instead of troponin T, as it is more specific to myocardial damage [4]. However, in cases with ambiguous symptoms or non-specific/unremarkable ECG findings, other causes of troponin elevation must be taken into consideration. Troponin elevation can occur from both coronary and non-coronary etiologies.

As this case study illustrated, high CK levels together with the immunological panel raised the suspicion of an inflammatory myopathy. CK is an enzyme used to release and transport energy, found in a variety of tissues within the body. While the skeletal muscles regenerate, it has been shown that there is an increase of CK levels in the new skeletal muscles, leading us to further investigate skeletal muscle abnormalities.

Previous literature

Several case reports have documented elevated troponin levels in patients with inflammatory myopathies such as dermatomyositis with absent cardiac symptoms. In such cases, patients often present with progressive muscle weakness and elevated muscle enzymes, leading to diagnostic uncertainty. Similar to this case, these reports highlight the challenges faced in distinguishing cardiac and non-cardiac causes of troponin elevation, especially in patients with unremarkable ECGs and no clear ischemic changes or symptoms. In a review of inflammatory myopathies and their cardiac manifestations, studies found that patients with inflammatory myopathies may exhibit elevated cardiac biomarkers, even when there are no overt signs of coronary artery disease. These cases underscore the need for clinicians to consider inflammatory myopathies as potential causes of elevated troponin levels, particularly in patients with muscle weakness and elevated CK [4].

Inflammatory myopathy

Inflammatory myopathies, including but not limited to dermatomyositis and polymyositis, are a group of autoimmune disorders characterized by chronic muscle inflammation, leading to muscle weakness and elevated muscle enzymes. Dermatomyositis is distinguished by its characteristic skin involvement presenting as a rash and perifascicular atrophy with perivascular, perimysial, and endomysial inflammation and can involve multiple organ systems, including the heart, lungs, and joints. Cardiac involvement, though less common, can present as myocarditis (inflammation of the myocardium), arrhythmias, or elevated troponin levels [4].

## Conclusions

This case sheds light on the complexity of diagnosing causes of elevated troponin levels. With the emphasis that elevated troponin is not always synonymous with ACS. We should approach such cases as a whole, taking the immune panel, raised CK, and skin findings, to help direct the physicians In this patient. Troponin elevation was ultimately attributed to dermatomyositis rather than ACS or NSTEMI. Post-steroid therapy made a significant improvement in the patient's symptoms making an example of appropriate management plans when a correct diagnosis is reached. The case underscores the importance of a comprehensive clinical assessment, considering the non-coronary causes in patients with elevated troponin, especially when ECG findings are unremarkable. Physicians must remain vigilant to avoid unnecessary invasive testing by thoroughly evaluating alternative causes such as inflammatory myopathies. Understanding the multifactorial nature of troponin elevation is of utmost importance to accurately diagnose and set the proper management plan.
